# MSC-Derived Extracellular Vesicle-Delivered L-PGDS Inhibit Gastric Cancer Progression by Suppressing Cancer Cell Stemness and STAT3 Phosphorylation

**DOI:** 10.1155/2022/9668239

**Published:** 2022-01-18

**Authors:** Benshuai You, Can Jin, Jiaxin Zhang, Min Xu, Wenrong Xu, Zixuan Sun, Hui Qian

**Affiliations:** ^1^Zhenjiang Key Laboratory of High Technology Research on Exosomes Foundation and Transformation Application, School of Medicine, Jiangsu University, Zhenjiang, 212013 Jiangsu, China; ^2^Department of Gastroenterology, Affiliated Hospital of Jiangsu University, Zhenjiang, 212013 Jiangsu, China; ^3^NHC Key Laboratory of Medical Embryogenesis and Developmental Molecular Biology & Shanghai Key Laboratory of Embryo and Reproduction Engineering, Shanghai 200040, China

## Abstract

Mesenchymal stem cell- (MSC-) derived extracellular vesicles (EVs) serving as delivery system have attracted extensive research interest, especially in cancer therapy. In our previous study, lipocalin-type prostaglandin D2 synthase (L-PGDS) showed inhibitory effects on gastric cancer growth. In this study, we aimed to explore whether MSC-EV-delivered L-PGDS (EVs-L-PGDS) could inhibit gastric cancer progression. EVs-L-PGDS were generated from MSCs transfected with adenovirus encoding L-PGDS. Cell colony-forming, migration, invasion, and flow cytometry assays were used to show the inhibitory effects of EVs on tumor cells in vitro, and the nude mouse subcutaneous tumor model was performed to show the inhibitory effect of EVs on tumor progression in vivo. In vitro, EVs-L-PGDS could be internalized and inhibit the colony-forming, migration, and invasion ability of gastric cancer cell SGC-7901 and promote cell apoptosis. In vivo, EVs-L-PGDS inhibited the tumor growth in nude mouse subcutaneous tumor-bearing model. Compared with the PBS and EVs containing empty vector (EVs-Vector) group, more apoptotic cells and higher L-PGDS expression were detected in tumor tissue of the EVs-L-PGDS treatment group. And these differences are significant. Mechanistically, EVs-L-PGDS reduced the expression of stem cell markers including *Oct4*, *Nanog*, and *Sox2* and inhibited STAT3 phosphorylation in gastric cancer cell SGC-7901. In conclusion, our results imply that MSC-derived EVs could be utilized as an effective nanovehicle to deliver L-PGDS for gastric cancer treatment, which provides a novel idea for the EV-based cancer therapy.

## 1. Introduction

Although the incidence of stomach cancer is generally declining, it has remained a heavy disease burden in developing countries over the past decades [1]. Despite advances in diagnosis and treatment, clinical outcome of advanced gastric cancer remains poor, and new therapeutic methods are urgently needed. Accumulating evidence has shown that mesenchymal stem cells (MSCs) hold promise for a wide range of applications in the treatment of many diseases including cancer [2, 3]. Tang et al. found that human umbilical cord MSCs (huc-MSCs) inhibited the growth of HepG2 cells and promoted their apoptosis [4]. huc-MSC-conditioned medium containing extracellular vesicles (EVs) could effectively induce apoptosis and attenuate the migration of tumor cells, which has attracted increasing attention [5]. These findings point to the positive effects of MSCs in cancer therapy.

EVs, including both microvesicles and exosomes, are small particles secreted by many types of cells and play key roles in intercellular communication [6, 7]. EVs from various origins hold great potential in cell-free anticancer treatment [8]. For example, tumor-derived EVs contain the similar components to those of the parent cell, indicating that EVs might target cancer sites [9]. Chemotherapy drugs and miRNA-134 and TNF-related apoptosis inducing ligands (TRAIL) could be delivered via tumor-derived EVs, thereby increasing the toxicity and targeting ability of these therapeutic reagents [10–12]. Dendritic cell-derived EVs could exhibit immunostimulatory characteristics, including inducing CD8^+^ CTL responses in cancer therapy [13, 14]. HEK239T cell-derived EVs are frequently used to achieve targeted delivery to tumor site by genetic modification on ligands [15–17]. MSC-derived EVs are also potential candidates for cancer treatment.

MSC-derived EVs have unique advantages as carriers for anticancer therapy [18]. Many studies have shown that MSCs hold the characteristic of tumor tropism, and EVs are able to carry a variety of biological active molecules from parent cells [19, 20]. Naseri et al. reported that EVs derived from MSCs could migrate to the tumor sites, which is similar to the ability of MSCs [21]. Besides, the immunogenicity of EVs is lower than that of MSCs due to a low amount of membrane proteins such as major histocompatibility complex (MHC) [22]. In addition, MSCs are highly proliferative and produce a large number of EVs under suitable culture conditions, which provides the feasibility for further clinical application [23]. Liu et al. found that MSC-derived extracellular vesicles transmitting miR-34a-5p could suppress tumorigenesis of colorectal cancer [24]. The therapeutic molecules carried by MSC-derived EVs are able to overcome the shortcomings of targeted tumor therapy [25]. These studies highlight the positive role of MSC-derived EVs as therapeutic molecular delivery carriers.

Lipocalin-type prostaglandin D2 synthase (L-PGDS) is the rate-limiting enzyme for the synthesis of prostaglandin D2 (PGD2), which catalyses the isomerization of prostaglandin H2 (PGH2) to PGD2 [26]. Studies have shown that PGD2 plays an important role in regulating physiological sleep and inducing allergic reactions [27, 28], as well as inhibiting tumorigenesis and development [29]. However, the half-life of PGD2 is short because of the presence of prostaglandin F (PGF) synthase and spontaneous dehydration in plasma, which make it limited for direct clinical application [30]. L-PGDS was involved in cyclooxygenase-2- (COX-2-) mediated apoptosis induction after chemotherapeutics [31]. L-PGDS deficiency resulted in decreased apoptosis of tumor cell, and L-PGDS-derived PGD2 was involved in antitumor responses [32]. Furthermore, in our previous study, PGD2/PTGDR2 signaling was found to be involved in regulating self-renewal and tumorigenesis of gastric cancer [29]. Overall, these studies indicate a potential promise of L-PGDS in anticancer therapy.

The promising roles of MSC-derived EVs are highlighted in multiple disease model treatment including cancer therapy. Therefore, the combination of EVs and therapeutic molecules may be a new direction for cancer therapy. In this study, we prepared EVs from huc-MSCs transfected with adenovirus encoding L-PGDS (EVs-L-PGDS) and aimed to evaluate the anticancer effect of the L-PGDS-loaded EVs on gastric cancer. Collectively, we demonstrated that EV-based delivery system is a novel strategy for cell-free cancer therapy.

## 2. Materials and Methods

### 2.1. Isolation of Human Umbilical Cord MSCs

huc-MSCs were isolated and identified as previously described [33]. Briefly, fresh umbilical cords were collected from informed, consenting mothers and rinsed twice with phosphate-buffered saline (PBS) containing penicillin and streptomycin, the cord blood being removed during this process. The washed cords were cut into 1 mm^2^-sized pieces and floated in Minimum Essential Medium *α* (*α*-MEM, Invitrogen) containing 10% FBS (Gibco), penicillin, and streptomycin. Tissues were then cultured at 37°C in a humidified 5% CO_2_ air condition. The medium was replaced every 3 days after the initial plating. When fibroblast-like cells appeared after 10 days, the culture was trypsinised and passaged to a new culture flask for further expansion, and the medium was changed every 2 days. The MSCs were subjected to osteogenic and adipogenic differentiation analysis and flow cytometric analysis of CD105, CD29, CD73, CD11b, CD34, and CD45 to confirm the successful isolation of MSCs. All cells were cultured at 37°C in a humidified 5% CO_2_ air condition.

### 2.2. Generation of EVs-L-PGDS

Adenovirus was purchased from Fubio Biological Technology Corporation. A schematic representation of the adenovirus vector is presented in Supplementary Figure 1. Adenovirus encoding L-PGDS (Ad-L-PGDS) and vector (Ad-Vector) were added to the MSC medium at 10^7^ PFU/ml when huc-MSCs reached 60–70% confluence. The transfection efficiency was observed by a fluorescence microscope. After 24 hours of transfection, the culture media were replaced with 8 ml of EV-free FBS/*α*-MEM for additional 48 hours. Then, the conditioned medium was collected for EV isolation. Briefly, the conditioned medium was centrifuged for 20 min at 2000*g* to remove dead cells, and subsequently, supernatant was centrifuged for 30 min at 10000*g* to remove cellular debris. Then, the clarified supernatant was concentrated and centrifuged for 30 min at 1500*g* using a 100 kDa MWCO hollow fiber membrane (Millipore, Bedford, MA); after that, the supernatant was fully resuspended with the ExoQuick-TC™ (SBI, EXOTC10A-1) and kept at 4°C overnight. EVs were acquired through centrifugation for 30 min at 1500*g* and stored at -80°C. The protein concentration of EVs was determined by BCA protein assay kit. The particle distribution of EVs was detected by nanoparticle tracking analyzers (Particle Metrix ZetaView®).

### 2.3. EV Uptake

The human gastric cancer cell line SGC-7901 was purchased from the Institute of Biochemistry and Cell Biology at the Chinese Academy of Sciences (Shanghai, China) and preserved in our laboratory and was maintained in RPMI-1640 medium with 10% FBS (Gibco). EVs were incubated with CM-Dil (Invitrogen) dye for 30 min at 37°C and washed twice using ultracentrifugation at 100,000 × *g* for 70 min to remove the excess dye. After that, SGC-7901 cells were incubated with the Dil-labeled EVs for 4 h, 4% PFA and 0.1% Triton-X 100 were used to fix and permeabilize the cells, and subsequently, the cell nuclei were stained using DAPI. Images were acquired using an inverted wide-field fluorescence microscope (Delta Vision Elite, GE Healthcare Life Sciences).

### 2.4. Cell Migration and Invasion Assays

SGC-7901 cells were seeded on a 6-well plate overnight and treated with PBS, EVs-Vector, and EVs-L-PGDS for 48 h. Then, 10^5^ cells were seeded into the upper chamber of Transwell chamber in serum-free medium and medium containing 10% FBS was added into the lower chamber and, subsequently, culturing in cell incubator for 16 hours. The chambers were fixed in 4% paraformaldehyde for 30 minutes and washed twice with PBS. After crystal violet staining and PBS cleaning, the number of migrated cells on the chamber surface was counted under the microscope, and at least six fields of cells were assayed for each group. For cell invasion assay, 200 *μ*l of Matrigel at a dilution of 1 : 4 in serum-free medium was precoated into Transwell chambers and cell incubation time was extended to 24 h. The remaining procedures were the same as those in cell migration assay.

### 2.5. Colony Formation Assay

SGC-7901 cells were harvested and seeded into a 6-well plate with the density of 1000 cells per well and incubated in a 5% CO_2_ humidified incubator at 37°C for 7 days. At the end of the incubation period, cells were fixed with 4% paraformaldehyde and stained with crystal violet. The number of cells was counted under the microscope.

### 2.6. Cell Apoptosis Analysis and TUNEL Staining

After treatment with PBS, EVs-Vector, or EVs-L-PGDS, SGC-7901 cells were harvested and stained with Annexin V and PI (Invitrogen) according to the manufacturer's instruction. The apoptotic cells were detected by flow cytometry. The apoptotic cells in tumor tissue were evaluated by TUNEL Apoptosis Detection Kit (Boster, Wuhan, China) according to the manufacturer's instructions. The positive cells were visualized and photographed with a light microscope.

### 2.7. Western Blot

Cell and tissue lysates were extracted in a lysis buffer (RIPA, Pierce) and proteinase inhibitor. Then, a total of 60 *μ*g protein was separated in 12% SDS-polyacrylamide gels with 180 kDa prestained protein marker (MP102-02, Vazyme Biotech Co., Ltd.) and transferred to PVDF membranes. The membranes were blocked with 5% BSA and then incubated with primary antibodies against GAPDH (1 : 2000; Bioworld), CD9 (1 : 500; Cell Signal Technology), CD63 (1 : 500; Abcam), CD81 (1 : 500; Abcam), Calnexin (1 : 500; Cell Signal Technology), L-PGDS (1 : 500; Bioworld), Bax (1 : 400; Cell Signal Technology), Bcl2 (1 : 500; Cell Signal Technology), Oct4 (1 : 500; Cell Signal Technology), Nanog (1 : 500; Bioworld), Sox2 (1 : 800; Wanlei bio), p-STAT3 (1 : 500; Cell Signal Technology), and t-STAT3 (1 : 500; Cell Signal Technology) at 4°C overnight. The membranes were washed three times with Tris-buffered saline/Tween and incubated with goat anti-rabbit secondary antibody (1 : 2000, Invitrogen) at 37°C for 1 hour. The signals were visualized using Luminata crescendo western horseradish peroxidase substrate (Millipore) and image software of GE (ImageQuant LAS4000 mini).

### 2.8. In Vivo Tumorigenicity

Xenograft mouse model was established as previously described [34]. Male BALB/c nu/nu mice (Laboratory Animal Center of Shanghai, Academy of Science, Shanghai, China) aged 4–6 weeks (18-20 g weight) were randomly divided into three groups (six mice per group). The mice were allowed free access to food and water and were housed at a controlled temperature (20–25°C) and humidity (50 ± 5%) on a 12 h light–dark cycle. All groups received subcutaneous injections of SGC-7901 cells pretreated with PBS, EVs-L-PGDS, or EVs-Vector at a protein concentration of 320 *μ*g/ml for 48 h (1 × 10^6^ cells in 200 *μ*l PBS) on side of the upper limbs. Tumor growth was evaluated using tumor weight and tumor volume measurement. Tumor volumes were measured using calipers according to the modified ellipsoidal formula (length × width^2^)/2. Tumors appeared on the 10th day and mice were sacrificed on the 25th day, and the harvested tumors were subjected to subsequent assays. All experimental protocols were approved by the Medical Ethics Committee of Jiangsu University (2012258).

### 2.9. Immunofluorescence and Immunohistochemistry

Immunofluorescence was used to detect the expression of L-PGDS in tumor tissue (1 : 50; Bioworld, Louis Park, MN). The secondary antibodies were Alexa Fluor 555-labeled donkey anti-rabbit IgG (1 : 300, Invitrogen, Carlsbad, CA). Images were acquired using microscopy (Nikon, Tokyo, Japan). For immunohistochemical analysis, the tumor tissues were fixed in 4% paraformaldehyde (PFA), embedded in paraffin, and cut into 5 *μ*m thick sections. Then, these sections were incubated with anti-PCNA (1 : 100; Bioworld, Louis Park, MN) overnight at 4°C and, subsequently, incubated with the secondary antibody at 37°C for 30 min. Finally, tissues were counterstained with 3,3′-diaminobenzidine (DAB) and photographed by microscopy.

### 2.10. Statistical Analysis

All data were shown as mean ± standard deviation (SD) and analyzed by GraphPad Prism software (version 7.0). The statistically significant differences between groups were assessed by one-way ANOVA with Tukey's post hoc test. *P* < 0.05 was considered statistically significant.

## 3. Results

### 3.1. Identification and Multipotency of huc-MSCs

**Figure 1:**
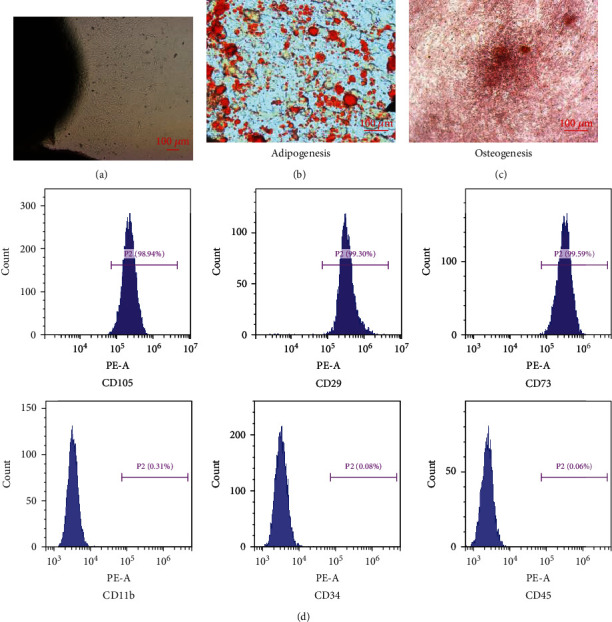
Identification and differentiation potential of huc-MSCs. (a) MSCs with their characteristic fusiform shape were grown on the 10th day of culture (×40). (b) Oil Red O staining for the adipogenic differentiation of huc-MSCs (×100). (c) Alizarin Red staining for the osteogenic differentiation of huc-MSCs (×100). (d) Flow cytometry for the surface antigens of huc-MSCs. The huc-MSCs expressed CD105, CD29, and CD73 but lacked expression of CD11b, CD34, and CD45.

huc-MSCs were obtained from fresh human umbilical cords. On the 10th day of culture, the huc-MSCs showed fibrous growth under the microscope (Figure 1(a)). Red fat droplets stained with Oil Red O were observed in huc-MSCs after being induced by adipogenic differentiation medium (Figure 1(b)). Red calcium nodules stained with Alizarin Red were observed in the osteogenic huc-MSCs (Figure 1(c)). Then, the surface markers of the huc-MSCs were detected by flow cytometry. The expression levels of CD105, CD29, and CD73 were positive, and the expression levels of CD11b, CD34, and CD45 were negative (Figure 1(d)). The above results indicate that we have successfully isolated huc-MSCs.

### 3.2. Ad-L-PGDS-Modified huc-MSCs Packaged L-PGDS into Secreted EVs

**Figure 2:**
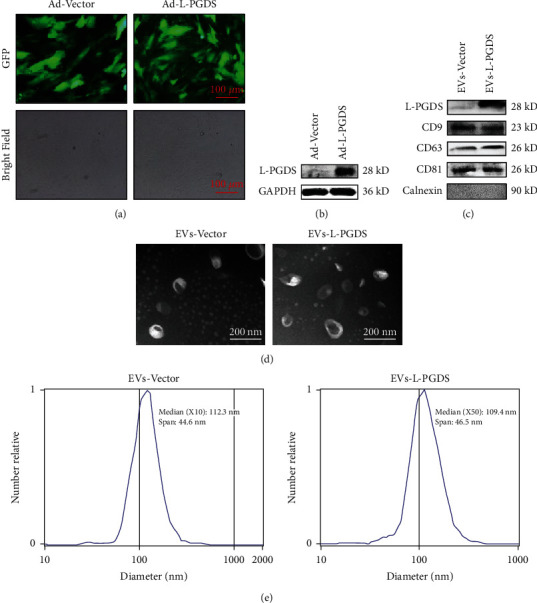
Ad-L-PGDS-modified huc-MSCs packaged L-PGDS into secreted EVs. (a) Representative fluorescence images of GFP in huc-MSCs after treatment with adenovirus for 24 h (×100). (b) Western blot for the expression of L-PGDS in adenovirus-transfected huc-MSCs after 24 h. (c) Western blot for the expression of EV markers CD9, CD63, and CD81 and the expression of L-PGDS in EVs. (d) TEM for the morphology of EVs. (e) NanoSight for the size of EVs. Abbreviations: TEM: transmission electron microscope.

Adenovirus encoding L-PGDS and vector were used to transfect huc-MSCs, respectively. The GFP expression in huc-MSCs was confirmed by a fluorescence microscope showing that the adenovirus was successfully integrated into the genome (Figure 2(a)). By staining the nucleus, almost all cells were successfully transfected with adenovirus, which showed the coexpression of DAPI and GFP (Supplementary Figure 2). Western blot showed that the expression of L-PGDS was significantly higher in Ad-L-PGDS-transfected MSCs than that in the Ad-Vector treatment group (Figure 2(b)). The supernatant of huc-MSCs transfected by virus was collected for EV isolation. The EVs secreted by huc-MSCs transfected with Ad-L-PGDS and Ad-Vector were marked as EVs-L-PGDS and EVs-Vector, respectively. Western blot showed that the EV markers, CD9, CD63, and CD81, were expressed in both EVs-L-PGDS and EVs-Vector (Figure 2(c)). Calnexin, a negative marker of EVs, was not expressed (Figure 2(c)). Compared with EVs-Vector, the expression of L-PGDS in EVs-L-PGDS was significantly increased (Figure 2(c)). Both kinds of EVs showed typical disc shape under the transmission electron microscope (TEM) (Figure 2(d)). In addition, the NanoSight visible nanoparticle analyzer detected that the particle distribution of EVs was relatively uniform, with a diameter of approximately 100 nm (Figure 2(e)). The results confirmed that L-PGDS-loaded EVs could be successfully generated by adenovirus transfection of MSCs.

### 3.3. EVs-L-PGDS Increased L-PGDS and Reduced Stem Cell Marker Expression and Inhibited STAT3 Phosphorylation in the Gastric Cancer Cell SGC-7901

**Figure 3:**
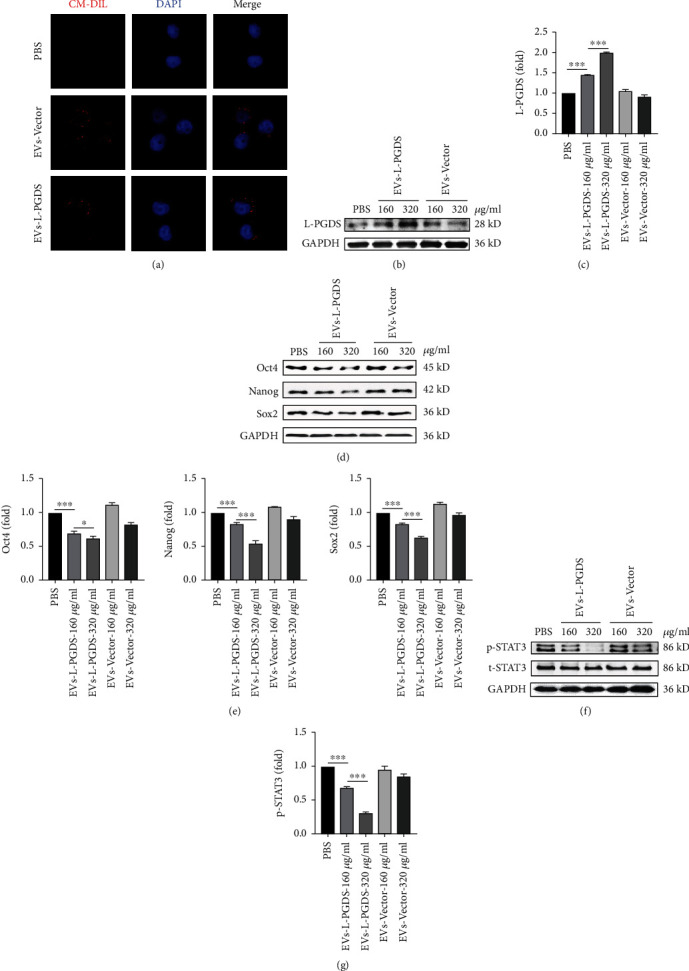
EVs-L-PGDS increased L-PGDS and reduced stem cell marker expression and inhibited STAT3 phosphorylation in the gastric cancer cell SGC-7901. (a) Confocal microscopy for the location of EVs in SGC-7901 cells (×600). (b) Western blot for the expression level of L-PGDS in SGC-7901 cells. (c) Quantitative analyses of protein expression of L-PGDS. (d) Western blot for the expression level of *Oct4*, *Nanog*, and *Sox2* in SGC-7901 cells. (e) Quantitative analyses of protein expression of *Oct4*, *Nanog*, and *Sox2*. (f) Western blot for the expression of p-STAT3 (Thy705), t-STAT3, and GAPDH in SGC-7901 cells. (g) Quantitative analyses of protein expression of p-STAT3 (Thy705). *n* = 3; ^∗^*P* < 0.05, ^∗∗^*P* < 0.01, and ^∗∗∗^*P* < 0.001.

The EVs were labeled with the lipid membrane dye CM-Dil and incubated with SGC-7901 cells to examine if they could be taken up by cancer cells. Fluorescence images showed the presence of red fluorescent spots in the cytoplasm near the nuclei of cancer cells via confocal microscopy, indicating the successful transfer of EVs into cancer cells (Figure 3(a)). Compared with PBS and EVs-Vector group, western blot showed that the expression of L-PGDS in SGC-7901 cells increased after EVs-L-PGDS treatment (Figures 3(b) and 3(c)). Consistent with our previous research, EVs-L-PGDS also reduced the expression of stem cell markers, including *Oct4*, *Nanog*, and *Sox2* compared with PBS and EVs-Vector group (Figures 3(d) and 3(e)). Besides, western blot analysis showed that the expression of phosphorylated STAT3 significantly decreased after EVs-L-PGDS treatment (Figures 3(f) and 3(g)).

### 3.4. EVs-L-PGDS Inhibited Migration, Invasion, and Colony Formation and Induced Apoptosis of Gastric Cancer Cell SGC-7901

**Figure 4:**
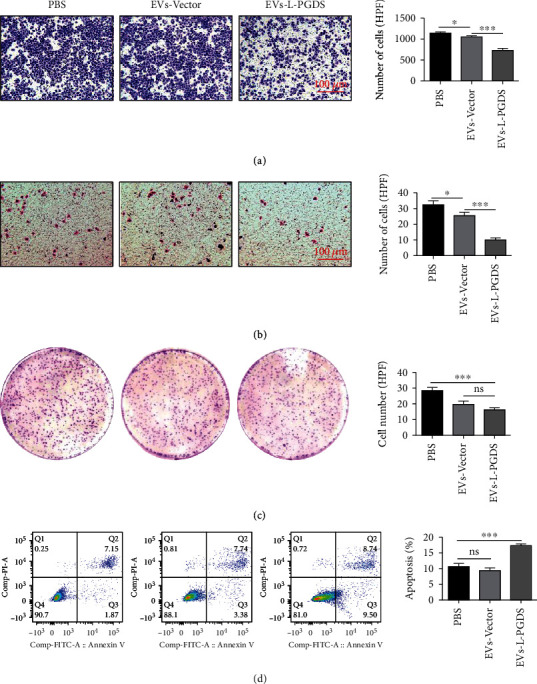
EVs-L-PGDS inhibited migration, invasion, and colony formation and induced apoptosis of gastric cancer cell SGC-7901. (a) Transwell migration experiments for the migration ability of SGC-7901 cells (×100). (b) Transwell invasion experiments for the invasion ability of SGC-7901 cells (×100). (c) Colony-forming assay for the colony formation ability of SGC-7901 cells. (d) Flow cytometry assay for the apoptosis level of SGC-7901 cells. *n* = 6; ^∗^*P* < 0.05, ^∗∗^*P* < 0.01, and ^∗∗∗^*P* < 0.001. Abbreviations: HPF: high-power field.

In order to investigate the effects of EVs on biological functions of cancer cells, EVs-L-PGDS and EVs-Vector were used to treat SGC-7901 cells. Compared with PBS, EVs-Vector had a slight inhibitory effect on the migration of tumor cells, while EVs-L-PGDS role in migration restriction was more effective (*P* < 0.05, PBS vs. EVs-Vector; *P* < 0.001, EVs-Vector vs. EVs-L-PGDS) (Figure 4(a)). EVs-L-PGDS also further inhibited the invasion ability of SGC-7901 cells (*P* < 0.05, PBS vs. EVs-Vector; *P* < 0.001, EVs-Vector vs. EVs-L-PGDS) (Figure 4(b)). In colony formation assays, the cancer cells treated with EVs-L-PGDS formed fewer colonies than the PBS group, while compared with the EVs-Vector group, the number of colonies did not change significantly, but the size was smaller (*P* < 0.001, PBS vs. EVs-L-PGDS) (Figure 4(c)). Flow cytometry analysis showed that the apoptosis rates in the PBS, EVs-Vector, and EVs-L-PGDS groups were 10.81 ± 1.62%, 9.50 ± 1.43%, and 17.62 ± 0.64%, respectively (Figure 4(d)). More apoptotic cells in the EVs-L-PGDS group were observed (*P* < 0.001, PBS vs. EVs-L-PGDS).

### 3.5. EVs-L-PGDS Inhibited the Growth of Subcutaneous Tumors in Nude Mice Induced by Gastric Cancer Cell SGC-7901

**Figure 5:**
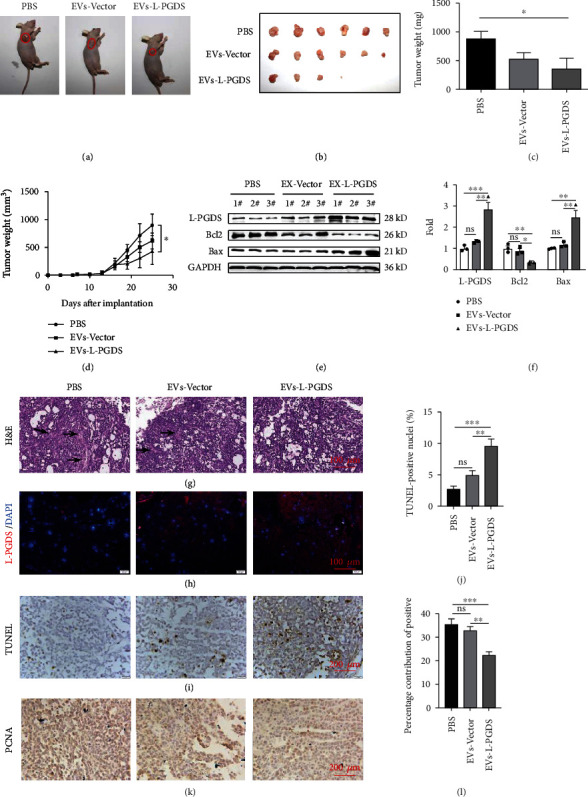
EVs-L-PGDS inhibited the growth of subcutaneous tumors in nude mice induced by gastric cancer cell SGC-7901. (a) Representative images of tumor-bearing mice. (b) Representative view of subcutaneous tumors. (c, d) Weight statistics and volume changes of tumor in mice treated with different EVs and PBS. (e) Western blot for the expression of L-PGDS, Bcl2, and Bax in tumor tissues. (f) Quantitative analyses of protein expression of tumor tissue. (g) HE staining of tumor tissues (×100). Arrowheads point to blood vessels. (h) Immunofluorescence staining for the expression of L-PGDS in tumor tissues (×100). (i) TUNEL assay for the apoptosis level in tumor tissues (×400). (j) Quantitative analyses of TUNEL-positive cells in tumor tissue. (k) Immunohistochemical staining for the expression of PCNA in tumor tissues (×400). (l) Quantitative analyses of PCNA expression in tumor tissue. *n* = 6; ^∗^*P* < 0.05, ^∗∗^*P* < 0.01, and ^∗∗∗^*P* < 0.001.

A subcutaneous tumor-bearing nude mouse model was performed to estimate the growth ability of SGC-7901 cells upon treatment with the EVs. Representative images of tumor-bearing mice are shown in Figure 5(a). Compared to another two groups, pretreatment with EVs-L-PGDS led to the production of smaller tumor mass (Figure 5(b)). The weight and volume of tumor tissue in the EVs-L-PGDS group were both the smallest (*P* < 0.05, PBS vs. EVs-L-PGDS) (Figures 5(c) and 5(d)). Immunofluorescence and western blot showed that more L-PGDS expression was found in the tumor tissue after EVs-L-PGDS treatment (Figures 5(e)–5(g)). Besides, the expression of Bax, a proapoptotic protein, was also significantly higher in the tumors of mice treated with EVs-L-PGDS compared with those treated with PBS (*P* < 0.01) and EVs-Vector (*P* < 0.01) (Figures 5(e) and 5(f)). And the expression of Bcl2 decreased in the EVs-L-PGDS group (*P* < 0.01, PBS vs. EVs-L-PGDS; *P* < 0.05, EVs-Vector vs. EVs-L-PGDS) (Figures 5(e) and 5(f)). HE staining demonstrated that the tumor tissue in the EVs-L-PGDS group was more porous and had less angiogenesis than that in the PBS and EVs-Vector groups (Figure 5(h)). TUNEL staining showed that there were more apoptotic cells in the tumor tissue in mice following treatment with EVs-L-PGDS compared with those treated with PBS (*P* < 0.001) or EVs-Vector (*P* < 0.01) (Figures 5(i) and 5(j)). Immunohistochemical examination of the expression of PCNA showed that the number of proliferating tumor cells was significantly lower in the tumors from mice in the EVs-L-PGDS group compared with the groups treated with PBS (*P* < 0.001) or EVs-Vector (*P* < 0.01) (Figures 5(k) and 5(l)). In short, our data suggested that L-PGDS-loaded EVs exhibited inhibitory effects on tumor growth in vivo.

## 4. Discussion

Our previous study found that L-PGDS expression was lower in gastric cancer tissues than in adjacent tissues and was associated with poor patient prognosis [29]. We have demonstrated that direct PGD2 stimulation or L-PGDS overexpression is able to inhibit gastric cancer cell growth and migration. Besides, Fukuoka et al. found that exogenous L-PGDS promoted PGD2 secretion of gastric cancer cells, thereby inhibiting the growth of gastric cancer cells by expressing peroxisome proliferator-activated receptor (PPAR *γ*) [35]. Furthermore, L-PGDS inhibited tumor growth better than PGD2 injection in tumor-bearing mice which prompted us to optimize the antitumor effects of PGD2 by carrying L-PGDS in EVs. Based on these studies, we hypothesized that MSCs could secrete EVs loaded with L-PGDS, and the modified EVs could restrict the progression of gastric cancer.

Emerging evidence suggests that EVs are useful delivery vehicles in tumor treatment due to their high stability, low immunogenicity, biocompatibility, and natural targeting ability [36, 37]. EVs contain abundant contents, such as mRNAs, proteins, miRNAs, and lipids; EVs could protect these cargos from degradation [38]. Over the past decades, researchers have identified different origin-derived EVs that could be utilized as vehicles to deliver anticancer drugs. Each type of EVs has distinct advantages. In this study, we used huc-MSCs as the origin of EVs. However, the roles of MSCs and MSC-derived EVs on cancer progression have been controversial. Some studies found favorable support of EVs for cancer progression. Roccaro et al. found that bone marrow MSC-derived EVs carry higher levels of oncogenic proteins, cytokines, and adhesion molecules to facilitate multiple myeloma progression [39], while other studies considered that EVs have therapeutic effects on cancer. For example, adipose MSC-derived EVs could inhibit the proliferation and induce apoptosis of ovarian cancer cells [40]. MSC-derived EVs were also shown to suppress hepatocellular carcinoma growth by promoting NK T cell antitumor responses [41]. Furthermore, engineering MSC-derived EVs via genetic or nongenetic methods enhances the antitumor effects [42, 43]. As to huc-MSCs, many studies have shown that huc-MSCs and their conditioned media containing EVs inhibit the growth and migration and induce apoptosis in many kinds of tumors including melanoma, lung cancer, hepatocellular carcinoma, and gliomas [5, 44, 45]. The reason for this discrepancy is unknown. One possible reason may be the timing of EV injection either before or after tumor formation [46, 47]. Another possible explanation for controversial results is due to the source of EVs. EVs may carry different molecules from their parent cells. For example, Zhu et al. showed that bone marrow MSC-derived EVs promoted gastric tumor growth in vivo [48]. Besides, the diverse effects of EVs on tumor progression may also be attributed to different tumor types [2, 49]. In this study, MSC-derived EVs containing empty vector also showed slight antitumor effects, indicating that huc-MSC-derived EVs are suitable candidates as anticancer drug carriers.

The cancer microenvironment contains a small subset of stem-like cells, which play important roles in cancer onset, maintenance, and metastasis [50]. The aggressive cancer cells usually have the enhanced potential in self-renewal ability, resulting in tumor progression. STAT3 is considered to have an important regulatory effect on the behavior of cancer stem-like cells [51]. By binding to the promoters of *Oct4*, *Nanog*, and *Sox2*, STAT3 could regulate the gene expression of some cancer stem-like cell markers, which may contribute to carcinogenesis and progression [52, 53]. In our previous study, we showed L-PGDS overexpression could restrict cancer cell stemness and suppress the activation of STAT3 [29]. In this study, our data indicated that the inhibition of STAT3 phosphorylation induced by EVs-L-PGDS is crucial for the expression of stem cell markers. Therefore, we speculate that EVs-L-PGDS may inhibit the gastric cancer progression by inhibiting STAT3 phosphorylation.

Various methods are employed to achieve EV modification. Incubation is often used to load chemotherapeutic drugs into EVs [54]. For loading miRNAs or siRNAs into isolated EVs, electroporation is the most commonly employed [55]. Most researchers use virus or plasmid transfection to genetically modify donor cells [56]. Herein, L-PGDS-loaded EVs were obtained via adenovirus-transfected huc-MSCs and characterized by TEM and western blot. We found that EVs-L-PGDS were internalized by cells, thereby inhibiting the colony formation, migration, and invasion and promoting apoptosis of SGC-7901 cells. Tumor growth was inhibited in SGC-7901 tumor-bearing mice after pretreatment with EVs-L-PGDS. These data indicate that adenovirus-transfected huc-MSCs are capable of secreting EVs enriched with therapeutic cargos and these cargos could be functionally delivered to tumor cells.

Before employing EVs, there are still many limitations. At present, there is no standard method for the isolation and extraction of EVs. Different methods including ExoQuick EV extraction kit, ultracentrifugation, microfluidic separation, and immunomagnetic bead sorting are developed for EV separation, which makes EVs present different qualities [57, 58]. Moreover, potential side effects and oncogenicity after long-term use should be mentioned and monitored in safety evaluations [59]. In addition, large-scale production of clinical-grade EVs is still difficult to achieve so far [60]. Additional efforts to these problems will improve the feasibility of clinical application of EVs.

In conclusion, our study demonstrates that EVs overexpressing L-PGDS inhibit gastric cancer progress by regulating cancer cell stemness and suppressing STAT3 phosphorylation. Our findings provide novel insights into the EV-based cancer therapy by genetic modification.

## Data Availability

The data used to support the findings of this study are included within the article.

## Supplementary Materials

Supplementary Materials Figure S1: schematic representation of the adenovirus vector. Figure S2: representative fluorescence images of GFP and DAPI in huc-MSCs after treatment with adenovirus. Supplementary Figure 3: uncropped western blot images for all figures.
