# Supplementary material for: MSC-Derived Extracellular Vesicle-Delivered L-PGDS Inhibit Gastric Cancer Progression by Suppressing Cancer Cell Stemness and STAT3 Phosphorylation
Source: Stem Cells Int. 2022 Jan 18;2022:9668239. doi: 10.1155/2022/9668239 (PMC8789473; doi:10.1155/2022/9668239)
Supplement: Supplementary Materials — Figure S1: schematic representation of the adenovirus vector. Figure S2: representative fluorescence images of GFP and DAPI in huc-MSCs after treatment with adenovirus. Supplementary Figure 3: uncropped western blot images for all figures. [file 9668239.f1.docx]

**Supplementary materials**

Title: MSCs-derived extracellular vesicles delivered L-PGDS inhibit gastric cancer progression by suppressing cancer cell stemness and STAT3 phosphorylation

Benshuai You^1^，Can Jin^1^，Jiaxin Zhang^1^ , Min Xu^2^, Wenrong Xu^1^，Zixuan Sun^1^*, Hui Qian^1,3^*

**Supplementary Fig1**

**
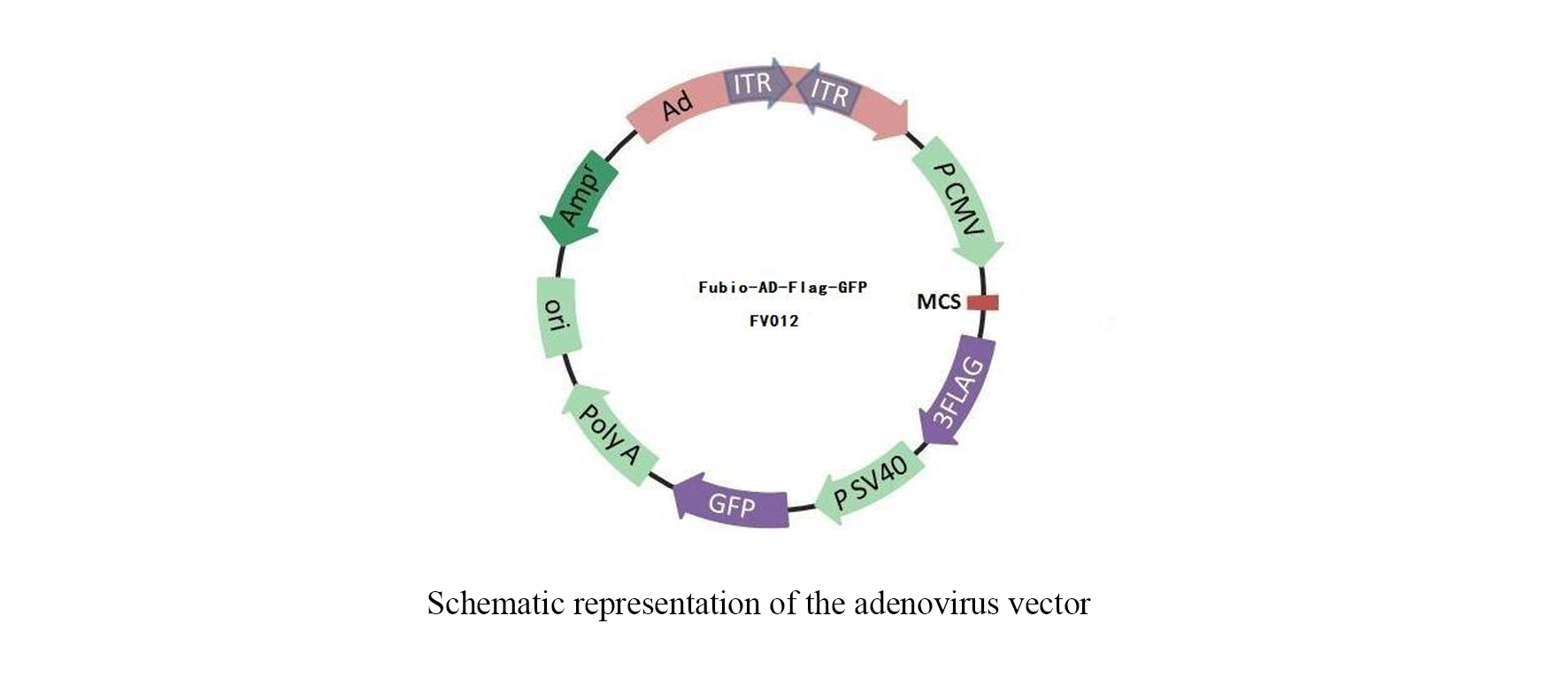
**

Supplementary Fig1. Schematic representation of the adenovirus vector

**Supplementary Fig2**

**
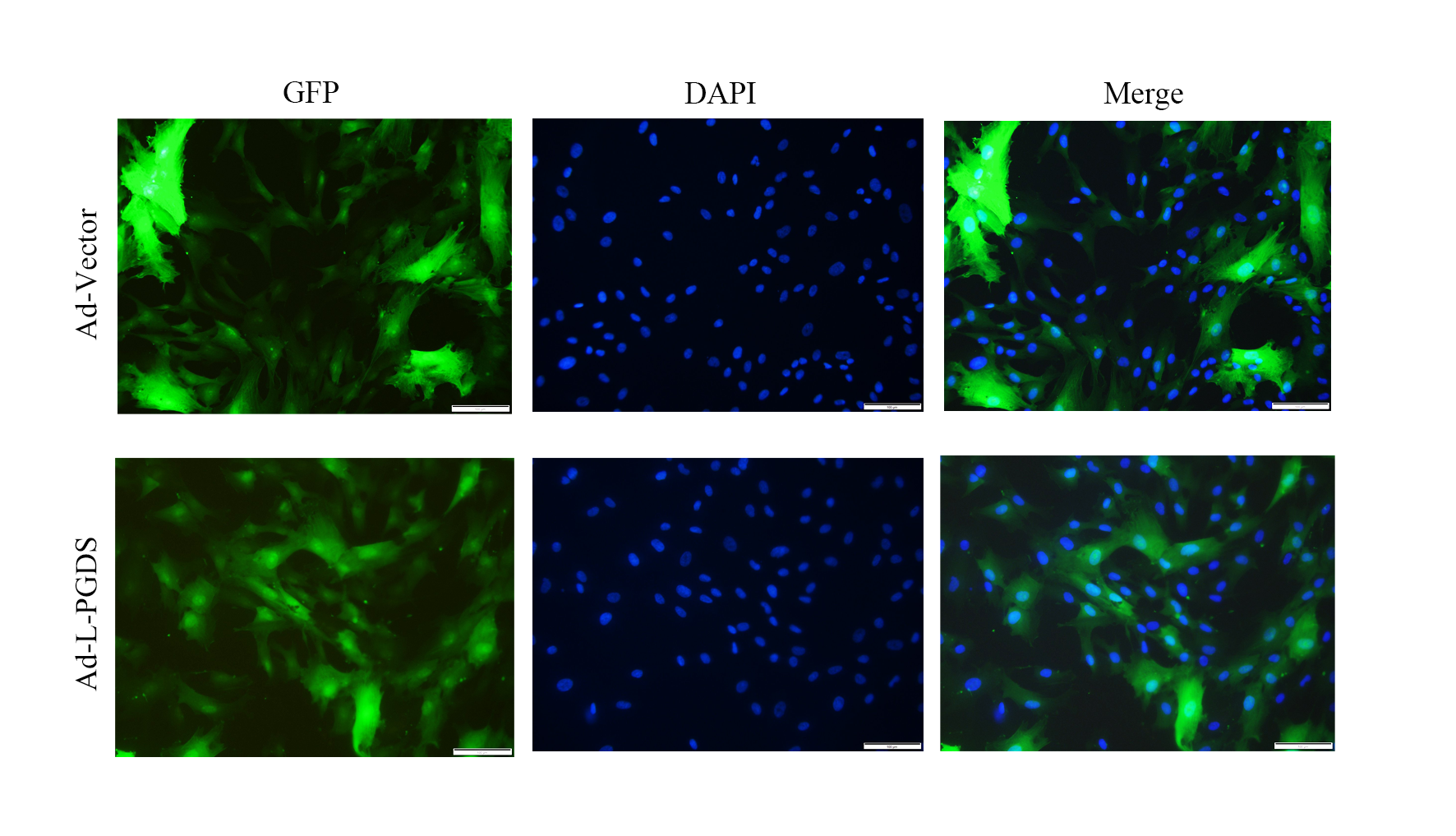
**

Supplementary Fig2. Representative fluorescence images of GFP and DAPI in huc-MSCs after treatment with adenovirus for 24h (100×). MSCs transfected with adenovirus were stained by DAPI.

**Supplementary Fig3**

**
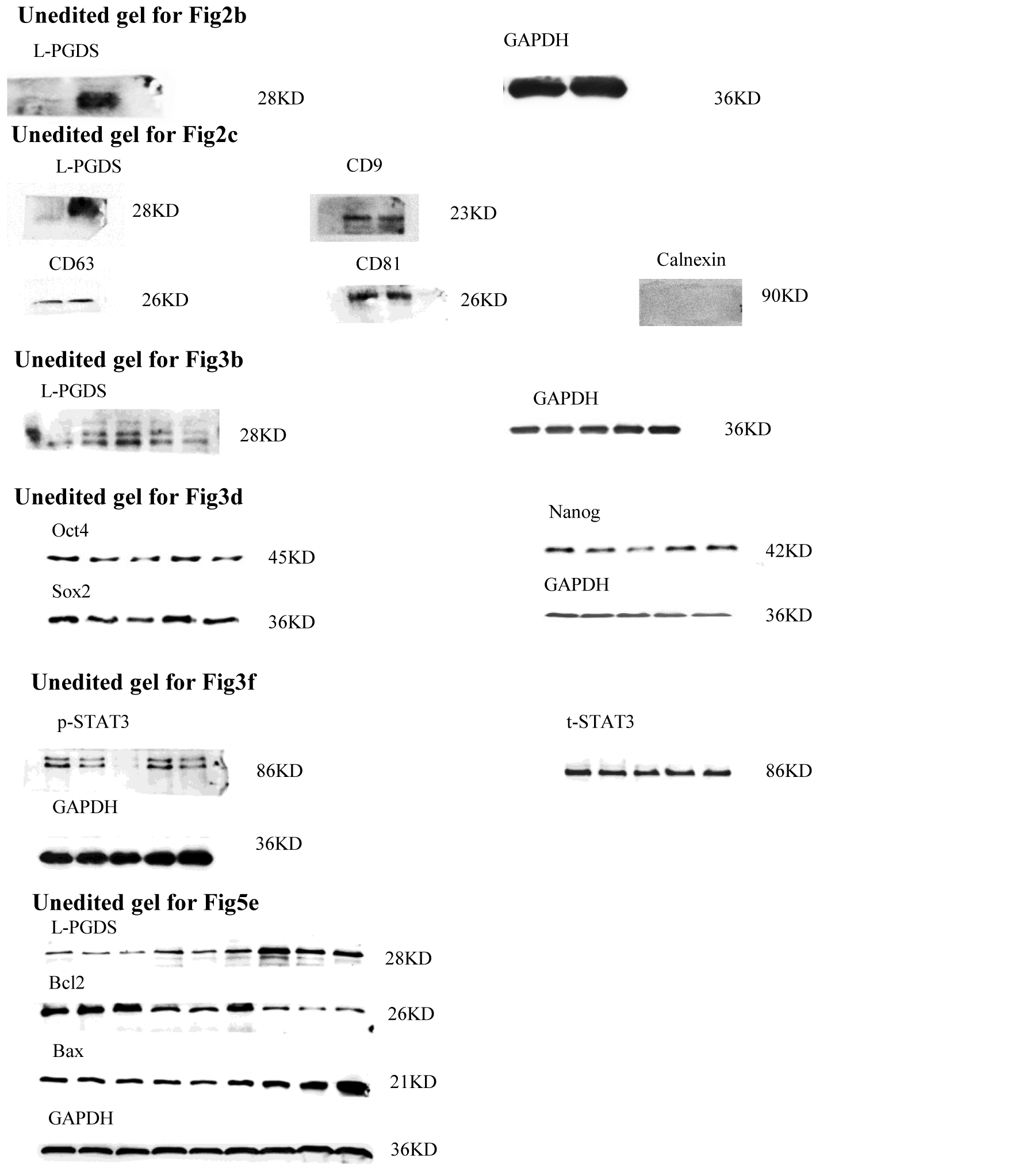
**

Supplementary Fig3. Uncropped western blot images for all figures.
